# The Asthma App as a New Way to Promote Responsible Short-Acting Beta2-Agonist Use in People With Asthma: Results of a Mixed Methods Pilot Study

**DOI:** 10.2196/54386

**Published:** 2024-04-04

**Authors:** Liselot N van den Berg, Cynthia Hallensleben, Lisa AE Vlug, Niels H Chavannes, Anke Versluis

**Affiliations:** 1 Department of Public Health and Primary Care Leiden University Medical Center Leiden Netherlands; 2 National eHealth Living Lab Leiden Netherlands

**Keywords:** asthma, short-acting beta2-agonist, SABA overuse, app, eHealth, feasibility, usability, mobile phone

## Abstract

**Background:**

Approximately 262 million people worldwide are affected by asthma, and the overuse of reliever medication—specifically, short-acting beta2-agonist (SABA) overuse—is common. This can lead to adverse health effects. A smartphone app, the Asthma app, was developed via a participatory design to help patients gain more insight into their SABA use through monitoring and psychoeducation.

**Objective:**

This pilot study aims to evaluate the feasibility and usability of the app. The preliminary effects of using the app after 3 months on decreasing asthma symptoms and improving quality of life were examined.

**Methods:**

A mixed methods study design was used. Quantitative data were collected using the app. Asthma symptoms (measured using the Control of Allergic Rhinitis and Asthma Test) and the triggers of these symptoms were collected weekly. Quality of life (36-Item Short-Form Health Survey) was assessed at baseline and after 3, 6, and 12 months. User experience (System Usability Scale) was measured at all time points, except for baseline. Furthermore, objective user data were collected, and qualitative interviews, focusing on feasibility and usability, were organized. The interview protocol was based on the Unified Theory of Acceptance and Use of Technology framework. Qualitative data were analyzed using the Framework Method.

**Results:**

The baseline questionnaire was completed by 373 participants. The majority were female (309/373, 82.8%), with a mean age of 46 (SD 15) years, and used, on average, 10 SABA inhalations per week. App usability was rated as good: 82.3 (SD 13.2; N=44) at 3 months. The Control of Allergic Rhinitis and Asthma Test score significantly improved at 3 months (18.5) compared with baseline (14.8; β=.189; SE 0.048; P<.001); however, the obtained score still indicated uncontrolled asthma. At 3 months, there was no significant difference in the quality of life. Owing to the high dropout rate, insufficient data were collected at 6 and 12 months and were, therefore, not further examined. User data showed that 335 users opened the app (250/335, 74.6%, were returning visitors), with an average session time of 1 minute, and SABA registration was most often used (7506/13,081, 57.38%). Qualitative data (from a total of 4 participants; n=2, 50% female) showed that the participants found the app acceptable and clear. Three participants stated that gaining insight into asthma and its triggers was helpful. Two participants no longer used the app because they perceived their asthma as controlled and, therefore, did not use SABA often or only used it regularly based on the advice of the pulmonologist.

**Conclusions:**

The initial findings regarding the app’s feasibility and usability are encouraging. However, the notable dropout rate underscores the need for a cautious interpretation of the results. Subsequent studies, particularly those focusing on implementation, should explore the potential integration of the app into standard treatment practices.

## Introduction

Asthma is a common chronic inflammatory disease, which is estimated to affect 262 million people worldwide [1]. Step 1 of medical treatment involves the prescription of short-acting beta2-agonist (SABA) as a reliever medication. In contrast to inhaled corticosteroids (ICS), SABA does not have an anti-inflammatory effect on the respiratory tract [2,3]. In 2019, step 1 was modified in the Global Initiative for Asthma guidelines [2]. Specifically, the option of a low dose of ICS-formoterol, as needed, was added because asthma control is often suboptimal [3-5]. According to guidelines, using SABA more than twice a week indicates suboptimal, uncontrolled asthma [3]. Approximately half of the patients with asthma have uncontrolled asthma [5-7]. The overuse of SABA is linked to an increased risk of asthma exacerbations, which are associated with damage to the respiratory tract, asthma-related hospitalization, and visits to the emergency department [8-12].

The overuse of SABA is common for different reasons. First, individuals often overuse their SABA instead of taking ICS to achieve a rapid relief from an asthma attack [13-15]. Second, individuals may lack knowledge about the medication and insight into the actual frequency of medication use [13,16]. For example, the REcognise Asthma and LInk to Symptoms and Experience study [17] found that 80% of the participants thought they had controlled asthma, although 40% had used their SABA ≥3 times during the past week. A post hoc analysis of the Dutch participants from this study showed that 60% of the patients with asthma overused their SABA in the previous week [18].

Previous studies have shown that self-management apps can help reduce the frequency of SABA use, increase SABA-free days, and improve overall asthma control [19,20]. These apps can also boost individuals’ confidence in managing asthma and improve their quality of life (QoL) [21-23]. Often, these self-management tools include education, self-monitoring, and feedback to support the end users in managing their disease daily [21,22,24,25]. Most apps are developed using state-based models, such as the Waterfall Model, and agile methods [26]. These traditional methods do not engage end users in the development process, which may result in lower usability and adherence of end users [27]. Therefore, an app was developed in collaboration with end users and other relevant stakeholders (eg, health care professionals) using a participatory design. This design can be used to engage relevant stakeholders during the development process, which may improve the usability and adherence to an app. The objective of the app is to help patients gain more insight into their SABA use while also promoting responsible SABA use. This may eventually decrease SABA overuse. In a previous study, we described the development process of the Asthma app [28].

This pilot study, using a mixed methods design, aims to examine (1) the feasibility and usability of the app in people with asthma and (2) the preliminary effects of using the app after 3 months on decreasing asthma symptoms and improving QoL.

## Methods

### Design and Population

The pilot study had a mixed methods design. Initially, the study was purely quantitative, with data collected through questionnaires administered in the app to examine the usability and preliminary effects of the app. Individuals were eligible to participate if they (1) were aged ≥18 years and (2) had asthma. Individuals who did not meet these inclusion criteria were excluded from the study; however, they could still use the app. The study period for the participants was 12 months. The study was conducted from January 15, 2021, to December 6, 2022; however, user data were collected until December 31, 2021. User data collection was stopped earlier because the costs for collecting these data increased after 2021, and this could no longer be funded.

During the study, we noticed that most participants used the app only in the first week after downloading. Owing to the high dropout rate, an additional qualitative study was conducted to examine the feasibility and usability of the app in more detail. Individuals who use, had used, or had downloaded the app once were included in the semistructured interviews. Individuals who participated in the qualitative interviews did not necessarily participate in the quantitative study. Qualitative interviews were held until data saturation was reached; data saturation was expected after 6 to 12 interviews [29,30]. Data were collected between November 7, 2022, and December 13, 2022.

### Ethical Considerations

According to the Medical Ethics Committee of the Leiden University Medical Center, this study did not fall within the scope of the Dutch Medical Research Involving Human Subjects Act (N20.103). Subsequently, a declaration of no objection was obtained from the Medical Ethics Committee. Participants provided informed consent and were able to opt out (see the *Procedure* section). The quantitative data were collected anonymously, and the qualitative data were collected pseudonymously.

### Asthma App

**Figure 1:**
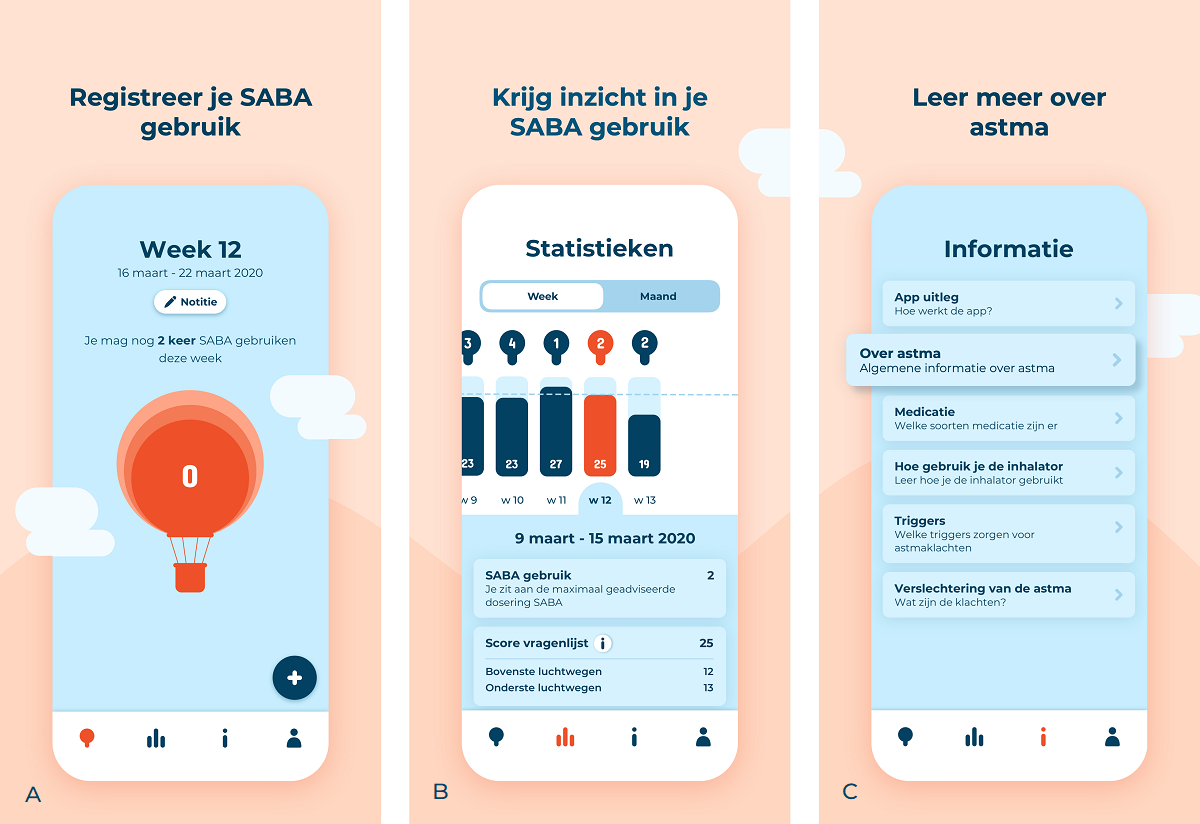
Visuals of the final version of the Asthma app: (A) landing page where users can register their short-acting beta2-agonist (SABA); (B) the graphical overview or statistics (in Dutch statistieken) where users can get insight into their SABA use, asthma symptoms, and asthma triggers; and (C) psychoeducation or information (in Dutch informatie) where users can learn more about their asthma and the app. On the landing page, users can receive three different messages based on the number of registered SABA compared with the prescription: (1) “You can still use your SABA # times this week,” (2) “You are at the maximum recommended dose of SABA for this week,” or (3) “You needed more SABA this week than advised.” After downloading the app, users receive an explanation on how to interpret the graphical overview, and this explanation can also be found in the informational part of the app.

The Asthma app (a Dutch app developed by the Leiden University Medical Center and Innovattic; Figure 1) allows end users to register their SABA use. Moreover, users can register asthma symptoms weekly (they receive a notification to do so), and they can register the triggers of these symptoms at any time. A graph shows how SABA use, asthma symptoms, and their triggers are related. The amount of SABA used was compared with the existing guidelines [2,3] or, when applicable, with health care professional’s advice. Psychoeducation is also included, covering topics such as *what is asthma* and *types of medication and their function* [28]. The app was available free of charge in the App Store and Google Play Store.

### Procedure

#### Quantitative Data

Different channels were used to announce the app’s go-live and to recruit participants. Relevant organizations (eg, Lung Foundation Netherlands and National eHealth Living Lab) posted the information on their website and social media, or only on their website or social media, and the closed Facebook group *Asthma and Peers* in the Netherlands published the information as well. The information was further communicated through publications (ie, via the COPD Asthma General Practitioners Advice Group in a magazine for pharmacists assistants and in a national newspaper in the Netherlands). Moreover, flyers were distributed via general practices.

After downloading and installing the app, individuals were asked 2 questions to determine their eligibility for the study (ie, whether they were aged ≥18 years and had asthma). Eligible individuals were given information about the study and could decide whether they wanted to participate by signing an informed consent form in the app. In the app, participants could view the informed consent form and withdraw from the study at any moment if they wanted to. If individuals chose to withdraw their consent, they could continue using the app.

Next, participants were asked to complete the demographic and clinical characteristics questionnaire and the baseline questionnaire about QoL (ie, 36-Item Short-Form Health Survey [SF-36] [31]) and intentions to change behavior (ie, a short version of the Theoretical Domains Framework [32]). Asthma symptoms were measured weekly using the Control of Allergic Rhinitis and Asthma Test (CARAT) [33,34]. The triggers of the asthma symptoms, such as dust mites and hay fever, were asked at the end of the CARAT, and the user could also enter additional triggers throughout the week. At 3, 6, and 12 months, user experience (ie, System Usability Scale [SUS]; [35]) and QoL were assessed. No compensation was provided for completing the questionnaire.

#### Qualitative Data

To gain more insight into the usability and experiences with the app, the following recruitment text was used: “NeLL is looking for (former) users of the Asthma app to get more insight into the usability and experiences with the app, during a one-time interview.” We recruited participants for the semistructured interviews via relevant organizations (eg, Asthma Association of the Netherlands and Davos and National eHealth Living Lab) that posted the information on their website and social media, or only on their website or social media; the information was also posted in the closed Facebook group *Asthma and Peers* in the Netherlands. To increase the interview response rate, participants were recruited via the personal channels of the researchers. When a participant was recruited via personal channels, the researcher did not conduct the interview.

Interested individuals could contact the researchers via email. Subsequently, 1 of the researchers (LNvdB and AEV) would contact them to determine whether they were eligible to participate (ie, aged ≥18 years, having asthma, and [at least] having downloaded the app). Eligible individuals interested in participating received the informed consent form via email. The participants could sign the informed consent form digitally via Castor (ie, a digital, secure research environment) [36]. After signing the informed consent form, the participants received an email invitation to schedule the semistructured interview. We aimed to enroll individuals who use the app and former users (ie, those who had at least downloaded the app).

An interview protocol was developed (Multimedia Appendix 1) based on the Unified Theory of Acceptance and Use of Technology (UTAUT) framework [37]. These interviews were conducted to better understand the perceived usability and feasibility. Interviews were conducted web-based via Microsoft Teams and lasted between 30 and 45 minutes. The participants received a gift card of 30 euros (US $31.2).

### Outcome Measures

#### Demographic and Clinical Characteristics

General information about the participants and their asthma was obtained, including gender, age (birth year), level of education, type of asthma, degree of asthma control, and type of medication. Multiple answers could be selected when answering the question about the type of asthma (ie, allergic asthma, nonallergic asthma, exercise asthma, severe asthma, and do not know) and medication (ie, SABA, ICS, long-acting beta2-agonist (LABA), ICS+LABA, do not know, and no medication use). Furthermore, the participants were asked whether they had received specific advice from their general practitioner on how much SABA they could use per week. When the participant had not received specific advice or did not know whether they had received specific advice, the existing guideline of a maximum of 2 SABA intakes per week was used. When the participants received specific advice from their general practitioner on their SABA use, they could indicate how much SABA they could use per week.

The question “How much SABA did you use last week?” was used as a baseline measure of SABA use. To examine whether an individual’s asthma was stable or unstable during the last week and differed from their average SABA use, an additional question was asked: “How much SABA do you use on average per week?”. In the app, individuals could register their weekly SABA use by clicking on the plus sign shown on the home screen.

#### The Intention to Change Behavior

The intention to change behavior was assessed using 3 items of the subscale “Intentions” of the Theoretical Domains Framework questionnaire [32]. The original subscale consisted of 4 items, but 1 of the items did not apply to this study and was, therefore, omitted. Items were answered on a 7-point Likert scale ranging from strongly disagree (1) to strongly agree (7). An example of an item is “In the next three months, I intend to use my SABA as prescribed.” A higher score (with a maximum of 21) signified more intent to use their SABA as prescribed in the next 3 months.

#### Feasibility and Usability

Different types of user data in the app were collected via an analytics platform (ie, PIWIK), namely, (1) which pages are visited in the app (ie, home screen, psychoeducation, user settings page, questionnaires, and the graph) and (2) events (ie, when the app is opened; SABA registrations; number of user clicks on notifications; and, when applicable, made changes in the maximum intake of SABA as advised by the health care professional).

The usability of the app was measured quantitatively using the 10-item SUS [35]. The items were rated on a 5-point Likert scale ranging from strongly disagree (0) to strongly agree (4). The scores were multiplied by 2.5 to obtain the total score ranging from 0 to 100. A higher score indicated that the app was more user-friendly.

A qualitative assessment of the feasibility and usability was conducted through interviews. The interview protocol was based on the UTAUT framework [37], which identified four main factors that influence the intention and use of technology (in this case, an app): (1) performance expectancy, (2) effort expectancy, (3) facilitating conditions, and (4) social influence. Textbox 1 presents an explanation of these factors. Moreover, the UTAUT framework includes four moderating factors: (1) gender, (2) age, (3) experience, and (4) voluntariness of use [37]. These factors and moderating factors were discussed during the interviews.

**Explanation of the factors within the Unified Theory of Acceptance and Use of Technology framework.**

**Description**

- Performance expectancy: the general benefits associated with app use and feasibility of the app
- Effort expectancy: ease of use and usability of the app
- Facilitating conditions: having sufficient resources and knowledge to use the app
- Social influence: the influence of other people (eg, family, friends, and acquaintances) to start and keep using the app and whether they would recommend the app to others

#### Preliminary Effects

Asthma symptoms and triggers of these symptoms were measured using the 10-item CARAT [33,34]. An example item is “During the last week, because of your asthma/rhinitis/allergy, how many times, on average, did you experience sneezing?.” Items were rated on a 4-point scale ranging from 0 (never) to 3 (almost every day). All items were reverse scored, and the total score ranged from 0 (minimal control) to 30 (maximum control). A score of 24 or higher indicated controlled asthma. In addition to the total score, 2 subscales were calculated: a score of the upper airway and a score of the lower airway. The upper airway score ranged from 0 (minimal control) to 12 (maximum control), and the lower airway score ranged from 0 (minimal control) to 18 (maximum control). An additional question was added to identify the symptom triggers: dust mites, animals, smoke, weather, hay fever or pollen, air pollution, smells, and exertion or exercise. Participants were able to select multiple triggers.

Participants’ health and health-related QoL were measured using the SF-36 [31]. The SF-36 consists of 2 main categories: physical and mental health [38]. Physical health entailed the physical components and consisted of the following subscales: physical functioning (10 items), role limitations due to physical problems (4 items), bodily pain (2 items), and general health perceptions (5 items). Mental health entailed the mental components and consisted of the following subscales: social functioning (2 items), general mental health (5 items), role limitations due to emotional problems (3 items), and vitality (4 items) [31,39]. All items were recoded into scores ranging from 0 (the poorest level of physical or mental health) to 100 (the best level of physical or mental health) [40], with higher scores indicating better health and higher QoL.

### Statistical Analysis

All quantitative data were analyzed using SPSS (version 25.0; IBM Corp) [41]. Descriptive analyses (eg, means, SDs, and percentages) were used to describe the demographic and clinical characteristics of the participants, intention to change behavior, user experience, QoL, and user data (eg, frequency of weekly SABA use). A mixed model was used to determine the change in asthma symptoms over time from the first week of using the app to 3 months after baseline. QoL at 3 months was compared with baseline data using Wilcoxon signed rank tests. The effects at 6 and 12 months were not examined because of the high dropout rate during the study period (88.2% at 3 months and more dropouts beyond that).

Interviews were audiotaped for subsequent analyses, and all audio records were transcribed intelligent verbatim by 1 researcher (AEV). Qualitative data analyses were performed by 2 researchers (LNvdB and AEV) according to the principles of the Framework Method [42] using Atlas.ti (version 22.0) [43]. The Framework Method is a systematic and flexible approach often used for the thematic analysis of semistructured interview data. Following transcription, the 2 researchers immersed themselves in the interviews to gain a comprehensive understanding. Subsequently, a deductive approach was adopted to code the interviews based on a predefined concept codebook developed beforehand based on the UTAUT framework [37]. The coding process was conducted independently by the 2 researchers, followed by a comparison of the codes. Additional codes were incorporated into the codebook, where applicable. A framework matrix was used to organize the data comprehensively, featuring relevant quotes from the participants. Finally, the characteristics and distinctions within the data set were identified. Throughout the process, the steps and data were discussed with the researchers CH and AV.

## Results

### Demographic and Clinical Characteristics

In the quantitative study, 485 individuals participated at baseline. Of these 485 individuals, 373 (76.9%) reported that they used SABA. Only these individuals were included in the analysis. Most of the participants were female (309/373, 82.8%) with a mean age of 46 (SD 15) years, had a secondary vocational education or higher (316/373, 84.7%), and had allergic asthma (187/373, 50.1%). At baseline, participants stated that they used, on average, 10 SABA per week and 10 SABA in the week before using the app. Moreover, the mean intention to change behavior was 17.1. This indicates that the participants wanted to use their SABA as prescribed for the next 3 months. Table 1 shows an overview of the demographic and clinical characteristics and the intention to change behavior.

**Table 1:** Baseline demographic and clinical characteristics of the participants and the intention to change behavior in the quantitative study.

Characteristic		Values
**Gender (n=373), n (%)**		
	Male	63 (16.9)
	Female	309 (82.8)
	Rather not say	1 (0.3)
Age^a^ (y; n=371), mean (SD; range)		46.1 (15; 18-81)
**Educational level (n=373), n (%)**		
	Primary school	7 (1.9)
	Secondary education	50 (13.4)
	Secondary vocational education	136 (36.5)
	Higher professional education	121 (32.4)
	University education	59 (15.8)
**Type of asthma^b^ (n=373), n (%)**		
	Allergic asthma	187 (50.1)
	Nonallergic asthma	126 (33.8)
	Exercise asthma	164 (44)
	Severe asthma	104 (27.9)
	Do not know	31 (8.3)
**Self-reported asthma control (n=373), n (%)**		
	Good control	134 (35.9)
	Insufficient control	151 (40.5)
	Do not know	86 (23.1)
**Medication type used^b^ (n=373), n (%)**		
	SABA^c^^,d^	373 (100)
	ICS^e^	198 (53.1)
	LABA^f^	150 (40.2)
	ICS+LABA	127 (34)
	Do not know	0 (0)
	No medication use	0 (0)
Average SABA use in the last week: self-reported (n=373), mean (SD; range)		10.5 (12.6; 0-60)
Average SABA use per week: self-reported (n=373), mean (SD; range)		9.7 (11.6; 0-60)
**Had medication advice from the health care professional (n=373), n (%)**		
	Yes	246 (66)
	No	110 (29.5)
	Do not know	17 (4.6)
Average maximum prescribed SABA^g^ (n=246), mean (SD; range)		22.2 (16.9; 0-60)
Intention to change behavior (n=373), mean (SD; range)		17.1 (4.5, 3-21)

^a^The birth year of 2 participants was missing. These participants were excluded from the calculation of the mean age.

^b^Participants were able to select multiple answers.

^c^SABA: short-acting beta2-agonist.

^d^51 participants only used SABA and no other inhalers.

^e^ICS: inhaled corticosteroids.

^f^LABA: long-acting beta2-agonist.

^g^The maximum number of SABA inhalations per week, as prescribed by the participant’s health care professional.

In the qualitative part of the study, among the 6 to 12 participants that we planned to recruit, only 4 participants could be included and interviewed. Half of the interviewed participants were female (2/4, 50%), with a mean age of 55 (range 21-78) years. One participant completed senior general secondary education, 1 completed secondary vocational education, and 2 had higher professional education. Two participants stated that they still used the app: they had both been using it for 1 year and 5 months. Regarding *social influence* from the UTAUT framework [37], the app was recommended by the hospital to one participant, and the other participant found it via the asthma association. Two participants stated that they no longer used the app but had used it for approximately 1 or 2 weeks. They both started using the app after the recommendation from a family member.

### Feasibility and Usability

**Figure 2:**
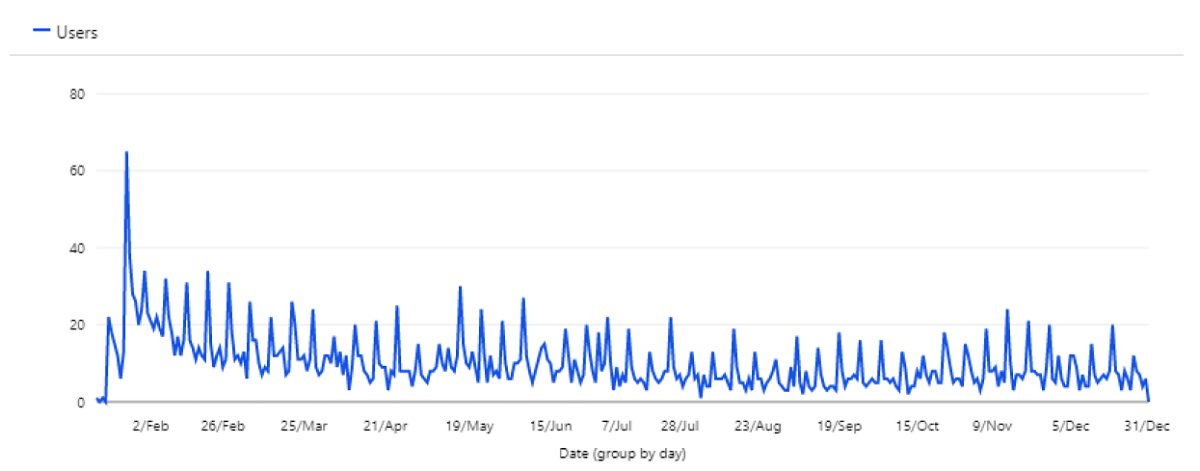
Number of users during the study period (January 15, 2021, to December 31, 2021).

User data showed that 335 unique users opened the app, of which 250 (74.6%) were returning visitors, with an average session time of 1 minute. An overview of the number of users during the study period is shown in Figure 2.

**Figure 3:**
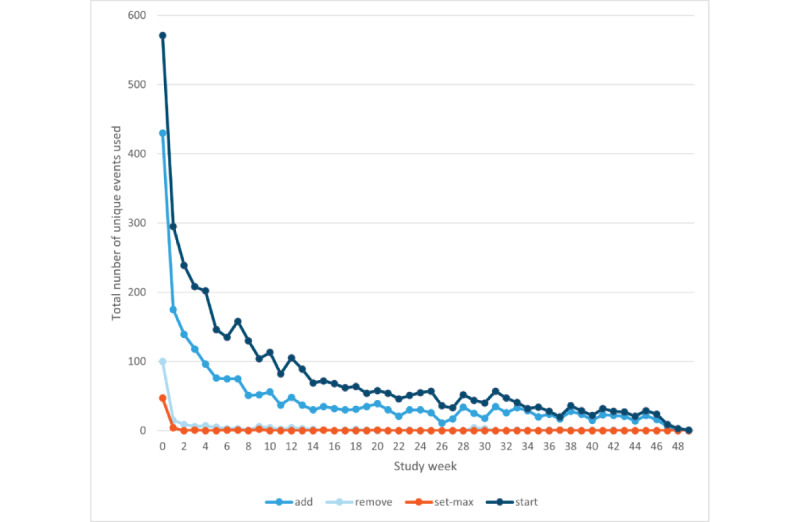
Unique events used per week: “add” means registering short-acting beta2-agonist (SABA), “remove” means removing a SABA registration, “set-max” means changing the maximum amount of SABA, and “start” means starting the app after giving informed consent and filling in the first questionnaires.

**Figure 4:**
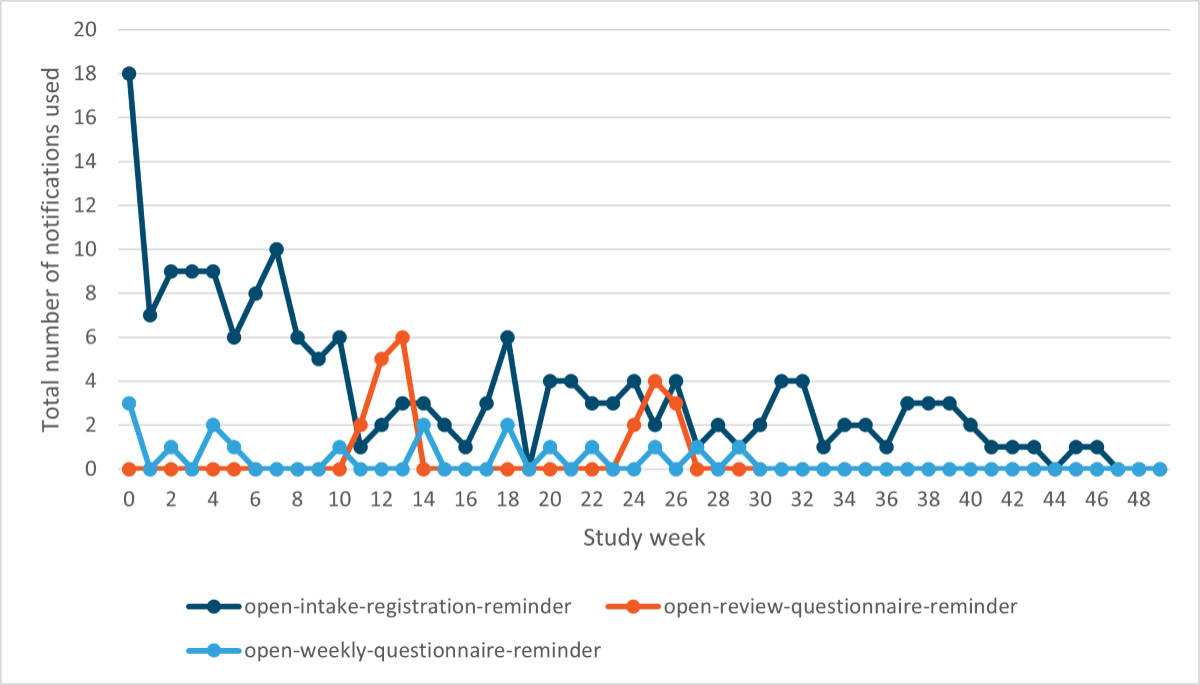
Events used per week, whether participants opened the app via 1 of the notifications: “open-intake-registration-reminder” is the notification users received when they did not register any short-acting beta2-agonist before the end of the week (Sunday); “open-review-questionnaire-reminder” is the notification for the questionnaires used at 3, 6 and 12 months; and “open-weekly questionnaire-reminder” is the notification for the weekly Control of Allergic Rhinitis and Asthma Test.

Most users opened the app via their smartphone (303/335, 90.4%), followed by a tablet (27/335, 8.1%) and a phablet (4/335, 1.2%). On average, the users had 5 events (ie, starting the app, adding SABA, removing SABA, changing the maximum amount of SABA, and clicking on 1 of the notifications) per session. Registration of SABA (ie, add-function) was most often used (7506/13,081 times, 57.38%). An overview of the events used per week is shown in Figures 3 and 4. At 3, 6, and 12 months, users registered an average of 5 SABA intakes per week (Table 2).

**Table 2:** Registered short-acting beta2-agonist use per week.

Time point			Values, mean (SD; 95% CI)
**Baseline (n=373)**			
	Baseline questionnaire	10.5 (0.5; N/A^a^)	
**3 months (n=19)**			
	12 weeks after baseline	4.67 (2.5; 1.64-13.29)	
**6 months (n=11)**			
	25 weeks after baseline	4.82 (2.6; 1.68-13.81)	
**Latest time point^b^ (n=2)**			
	48 weeks after baseline	5.24 (3; 1.71-16.09)	

^a^N/A: not applicable.

^b^Measures ended within 1 year (mid-January 2021 until the end of December 2021).

Usability of the app, as assessed with the SUS, was good over the entire study period: 82.3 (SD 13.2; n=44) at 3 months, 84 (SD 13.6; n=26) at 6 months, and 82.3 (SD 13.4; n=11) at 12 months (Table 3).

**Table 3:** Questionnaire results regarding usability and quality of life.

Questionnaire			Baseline		3 months		6 months		12 months
**Usability**									
	Values, n (%)	N/A^a^		44 (100)		26 (100)		11 (100)	
	Values, mean (SD)	N/A		82.3 (13.2)		84 (13.6)		82.3 (13.4)	
**Quality of life: physical health**									
	Values, n (%)	373 (100)		44 (100)		26 (100)		11 (100)	
	Values, mean (SD)	53.6 (22.4)		56.1 (23.9)		57.6 (21.8)		54.9 (24.7)	
**Quality of life: mental health**									
	Values, n (%)	373 (100)		44 (100)		26 (100)		11 (100)	
	Values, mean (SD)	57.4 (21.2)		62.9 (22.3)		59.4 (20.1)		67.8 (18.1)	

^a^N/A: not applicable.

Qualitative data showed that 3 (75%) of the 4 participants had experience using other health apps. The users mentioned that they wanted to use health apps that were fun and useful:

[...] I only want apps which I like or which are useful.

Male user, 78 years old

The participants found the app acceptable and clear in terms of *performance expectancy*. Three participants stated that gaining insight into asthma and its triggers was helpful. Another participant explained that it was not helpful at the moment because he considered his asthma to be controlled. This was for both participants who no longer used the app. One participant did not use SABA often, and the other participant only used it regularly based on the advice of his pulmonologist:

First impression was, well, I think, it looks clear. It was pretty clear to me on my own what I could do with it. After using it, yeah, I think it just looks like a nice app, not too old-fashioned. But just fairly new, as you expect from an app in this day and age. And it was also very quickly clear to me exactly what I could do with it.

Male user, 21 years old

As very useful; you open the app and click on the plus icon how many times if you use it at that time. And also very nice that you get a notification every now and then like, “hey, it is the end of the week; make sure you fill in the amount.” Especially if you forget to fill it in. That is nice.

Male user, 21 years old

Regarding *effort expectancy* and *facilitating conditions*, 3 participants stated that the app is easy to use and straightforward and does not require much effort to register SABA use. One of these participants also stated that the app was well written and easy to read. The fourth participant did not say anything about ease of use. However, 1 participant experienced difficulties in interpreting the questions and answering the possibilities of the CARAT:

So with a few questions, I got, well you already noticed that I have some difficulties with choosing the right one.

Male user, 78 years old

Of the participants who continue to use the app (2/4, 50%), they use it multiple times per week, with a minimum frequency of once per week and often 2 or 3 times per week. Opening the app was, for 1 participant, mostly completed after receiving a notification. A former user mentioned that he would use the app once a week to fill in all the SABA intakes for that week.

Multiple possibilities for improvement were mentioned during the interviews. One participant wanted to be able to fill in triggers that were not listed in the app and also wanted to have the possibility to add more types of medication. Another participant missed contact with other patients with asthma in the app to discuss, for example, medication use. Someone else would change the CARAT based on their experienced difficulties. The last participant missed more background information about SABA use and why SABA should not be used more than twice a week:

There is one question that I do not understand. I filled it in good conscience in, and it immediately gave a number that should be decisive, but that I think “yes, but this does not apply to me.” In the app, it asked “how many times a night do you wake up?” [...] I do wake up but with a different cause [...] I personally think, but that is my opinion, there should stand “Do you wake up at night, because of your asthma?

Male user, 78 years old

Further exploring *social influence* showed that all the participants would recommend the app to others because they experienced that it provided more insight into their medication use and they received more information about the complete picture of asthma. One participant had already recommended the app to an acquaintance, who also started using the app. The 2 former users would specifically recommend it to certain patients: people with severe asthma or uncontrolled asthma or people who do not take their reliever medication as intended. Furthermore, 3 participants also found it useful to show the app to their health care professional during a consultation:

I would recommend it, especially to people who do not really have a case like mine. I would also not recommend it to people who, like me, only use salbutamol for sports. Yes, I do not know those people who just do it for sports just like me. Then it does not make much sense to keep track of how often you use it. You just know how often you exercise, and if you first use salbutamol then you know “hey, I use it so often.” But for people who use it often, it seems to me that is a very handy app, especially if you can see in that graph how often you have used it per week and in which week more and in which week less.

Male user, 21 years old

### Preliminary Effects

At week 1, the mean CARAT score was 14.8. This indicated that the participants’ asthma was uncontrolled. Their CARAT score improved significantly to a mean score of 18.5 after 12 weeks (ie, 3 months; β=.189; SE 0.048; P<.001); however, this mean score still indicated that their asthma was uncontrolled. This was also the case for both the upper airway score, which significantly improved from a mean score of 6.8 to 7.7 after 12 weeks (β=.073; SE 0.027; *P*=.009), and the lower airway score, which significantly improved from a mean score of 8 to 10.8 after 12 weeks (β=.121; SE 0.037; *P*=.002).

The top three asthma triggers reported in week 1 were (1) weather (321/435, 73.8%), (2) exertion or exercise (305/435, 70.1%), and (3) smoke (197/435, 45.3%). After 12 weeks (ie, 3 months), the top three triggers were (1) weather (25/37, 68%), (2) exertion or exercise (19/37, 51%), and (3) hay fever or pollen (17/37, 46%).

At 3 months, there was no significant difference compared with baseline regarding the mean physical and mental health scores (*Z*=−0.074; *P*=.94 and *Z*=−0.117; *P*=.91, respectively; Table 3).

## Discussion

### Principal Findings

This study aimed to determine the feasibility and usability of a newly developed app, the Asthma app. Furthermore, the preliminary effects of using the app after 3 months on decreasing asthma symptoms and improving QoL were examined. The quantitative data showed that the usability was good. This was also found in the qualitative data: the app was considered easy to use, and it did not take much effort to register SABA. Furthermore, most participants stated that the app was useful for gaining insight into asthma, triggers, and medication use, and therefore, the app was considered feasible and usable. Multiple improvement possibilities were mentioned during the interviews, such as adding additional personal triggers next to the existing standard list of triggers and the availability of a social support network to contact others with asthma easily. In addition, former users, who no longer used the app, stated that they would recommend the app to people with severe asthma or uncontrolled asthma or people who do not take their reliever medication as intended.

As for the preliminary effects, an improvement in asthma symptoms was found after 3 months; however, the mean asthma symptom score still indicated that the asthma was uncontrolled. Improvement in asthma symptoms was also found in other eHealth studies [19,20]. The mean asthma symptom score in our study, indicating uncontrolled asthma, could be explained by the low intensity and noninvasive nature of the intervention (eg, users could use the app whenever and how often they wanted). A systematic review [44] also found that asthma control did not significantly improve in other studies. They proposed additional well-designed studies to gather more robust findings on what is necessary to achieve optimal asthma control [44]. In terms of QoL, no significant improvement was observed after 3 months. No effect was observed because poor asthma control was associated with worsened QoL [45,46]. The average uncontrolled asthma scores at week 1 and 3 months after baseline can be related to the low QoL scores at the same time points. Moreover, a systematic review [47] demonstrated that eHealth interventions have an inconsistent impact on QoL in people with asthma. The systematic enhancement of clinical outcomes such as QoL was mostly observed within the whole-systems approach, taking into account patient, professional, and organizational elements.

The data from this study should be interpreted with caution because of the high dropout rate, which resulted in insufficient data for conducting analyses at 6 and 12 months. Although a high dropout rate is frequently seen in studies investigating digital applications, we envisioned that the dropout rate would be lower in this study, considering the participatory design process [28]. The dropout may be explained by the higher probability of dropout in people with chronic diseases when they are impacted physically and mentally by the condition [48]. Most of the participants in this study had uncontrolled asthma and, therefore, more symptoms throughout the day and night. This could have resulted in lower or no app use, which was directly linked to the withdrawal from the study. Another explanation for the high dropout rate could be, as described in our previous study [28], that only a minimal viable product was evaluated. Not all features recommended by the patients, such as registering additional controller medication, were implemented. Therefore, the app might not fit the needs of all the users and cause them to stop using the app.

Using the UTAUT framework [37], performance expectancy was positively associated with the use of the app for the current users. The app will help them gain more insight into asthma, triggers, and medication use. Performance expectancy was lower for former users who stated that their asthma was controlled; therefore, the aim of the app did not align with their needs. Effort expectancy was positively associated with both the intention to use and the actual use of the app, largely because of its user-friendly interface, minimal effort required for SABA registration, and language simplicity. The only aspect that was negatively related to the effort expectancy factor was difficulty with one of the questionnaires by a former user. Facilitating conditions were positively associated with the use of the app. The participants had the appropriate knowledge and resources to use the app. Technical support was not discussed during the interviews; however, clarity regarding the appropriate contact for technical issues could enhance user experience. Finally, social influence played an essential role in intention and use; all interviewees initiated app use through social media discovery or recommendations from health care professionals or family members. They would also recommend the app to others, and 1 participant had already recommended the app to an acquaintance. However, in future studies, this could be further explored in relation to voluntariness of use, which was not thoroughly explored in this study. This is also the case for other moderating factors such as *gender* and *age*. The sample size was too small to explore the associations between the moderating factors, factors, and intention and use of the app. Notably, prior experience with health apps positively influenced the intention and use of the app in this study, and current experience was positively influenced by effort expectancy, facilitating conditions, and social influence for current users.

### Strengths and Limitations

This study has several strengths and limitations. A notable strength was the use of a real-life setting for evaluating the Asthma app, allowing a comprehensive understanding of its feasibility and usability. In addition, interviews with both current and former users provided a nuanced perspective on user satisfaction and the factors influencing app use.

In addition to the previously mentioned high dropout rate, another limitation was that the questionnaires were exclusively offered in the app environment. Therefore, former users were no longer able to complete the study questionnaires, thus limiting the availability of their data at later time points (ie, after baseline). To obtain the perspectives of former users on feasibility and usability, they were included in the qualitative interviews. Nevertheless, the recruitment of this group was difficult, and only 2 former users could be included.

Finally, the intended target of 6 to 12 interviews to achieve data saturation [29,30] was not attained. This was partially attributed to the difficulty in reaching former users who may have lost interest in the app or study. Despite the small number of interviews conducted, similar findings were found during data collection between the 2 users and the 2 former users.

### Implications for Future Research and Practice

A minimal viable product was examined in this study. During the next development round, feedback gathered during the cocreation of the app could be re-evaluated [28], or new cocreation sessions could be organized to further enhance the app. In future studies, with a newer version of the app, the outcomes of this study could be further examined with more data at more time points, and clinical outcomes, such as the impact of the app on medication adherence, could be explored. A smart asthma inhaler [49,50] could also be linked to the app to gather real-time objective data instead of self-reported registration, which is more sensitive to biases.

This study has a high dropout rate. Renzi et al [51] stated in their review that reminders are often used to improve medication adherence in eHealth interventions but that this improvement is reduced over time. Typically, after 6 months, users tend to revert to their previous behaviors as the novelty of the eHealth intervention wanes [51]. This could also be the case in this study, especially because of the anonymous nature and the use of in-app questionnaires. In future studies, it would be advisable to collect data pseudonymously and send questionnaires via email to achieve a higher response rate. In this way, participants will also be less likely to withdraw from the study and stay involved for longer.

In the new version of the app, additional information about the treatment guidelines should be implemented, such as the fact that users should follow the advice from their health care professional if they receive any. It should be clarified that the app is specifically for people with asthma who only use SABA (and not ICS), which has been the first step of treatment for decades. Potential users could be reached via general practitioners, specialized practice nurses, or pharmacists when they prescribe or distribute SABA. Currently, the Asthma app is a stand-alone app, which means that it is used by patients without the involvement of health care professionals. However, involving health care professionals via “blended care” could improve the quality of care [52]. Moreover, health care professionals can offer additional education and guidance based on the data from the app [53]. To incorporate the app into standard treatment, it is necessary to develop a plan together with asthma associations and health care professionals. A designated implementation team can improve the success rate of the implementation [54], and it is important to explore context-specific strategies that align with the implementation process phase [55]. Certain barriers (eg, technical issues, time and attention requirements for use, low engagement from health care professionals, and shortage of funding) and facilitators (eg, stakeholder engagement and enthusiasm, minimizing workflow interruptions, and access to information about the app) should be taken into account when implementing the app in standard care [27,56-58]. In addition, more education about SABA overuse could make health care professionals more aware of the risks, which could prioritize the use of the app.

### Conclusions

This study evaluated the feasibility and usability of a new app for people with asthma. The initial results regarding usability were positive. Nevertheless, it is essential to exercise caution when interpreting these results because of the high dropout rate in this study. Two former users would recommend the app to people with severe asthma or uncontrolled asthma or people who do not use their reliever medication as intended. Future (implementation) studies could evaluate the potential of incorporating the app into standard treatment practices. Moreover, the actual impact of the app on clinical outcomes, such as medication adherence, should be further examined.

## Appendix

Multimedia Appendix 1 Semistructured interview protocol about the Asthma app.

## Abbreviations

CARAT: Control of Allergic Rhinitis and Asthma Test
ICS: inhaled corticosteroids
QoL: quality of life
SABA: short-acting beta2-agonist
SF-36: 36-Item Short-Form Health Survey
SUS: System Usability Scale
UTAUT: Unified Theory of Acceptance and Use of Technology
