# Supplementary material for: The Asthma App as a New Way to Promote Responsible Short-Acting Beta2-Agonist Use in People With Asthma: Results of a Mixed Methods Pilot Study
Source: JMIR Hum Factors. 2024 Apr 4;11:e54386. doi: 10.2196/54386 (PMC11027062; doi:10.2196/54386)
Supplement: Multimedia Appendix 1 [file humanfactors_v11i1e54386_app1.docx]

**Appendix 1**. Semi-structured interview protocol for people with asthma (in Dutch)

*Aim: With this interview, in addition to the questionnaires, we want to gain a more in-depth insight into the experience with, intention to use and actual use of the Asthma app.*

1. **Introduction, explanation and informed consent**
   1. Welcome: introduction interviewer and researcher
   2. Introduction topic
   3. Planning
   4. Informed consent
   5. Reimbursement
   6. Questions?
2. **Introduction round**
   1. Participant introduces him-/herself
3. **Opening topics**
   1. What are your prior experiences with health apps?
   2. What was your first impression of the Asthma app?
4. **Usage and experience Asthma app**
   1. How often do you use the Asthma app?
      1. When do/did you use the app?
      2. When do/did you not use the app?
   2. Why did you start using the Asthma app?
      1. *When not using the app*: What has been the reason to no longer use the Asthma app?
   3. Which features of the Asthma app have you used?
      1. *Note interviewer: the following features are part of the Asthma app: registering SABA, weekly questionnaire symptoms and triggers of these symptoms, overview (graph), psychoeducation, and profile (among other things changing maximum SABA usage).*
   4. How did you experience the usage of the Asthma app?
   5. Would you recommend the Asthma app?
      1. Why or why not?
   6. How could the Asthma app be further improved?
5. **The future**
   1. *When using the app:* Would you keep using the Asthma app in the near future?
      1. Why or why not?
   2. *When no longer using the app:* Would you start using the Asthma app in the near future?
      1. Why or why not?
6. **Closing**
   1. What is the most important topic we discussed today?
   2. Do you have additional comments/questions?
   3. Do you think differently about the Asthma app than before this interview?
   4. End of interview
